# Exploring South Korean Elementary School Classroom Teachers’ Beliefs and Practices in Physical Education

**DOI:** 10.3390/ijerph192215033

**Published:** 2022-11-15

**Authors:** Jongho Moon, Yongnam Park

**Affiliations:** 1 Department of Human Performance and Health Education, Western Michigan University, Kalamazoo, MI 49008, USA; 2 Naedong Elementary School, 426 Sunhwan-ro, Naedong-myeon, Jinju-si 52849, Gyeongsangnam-do, Republic of Korea

**Keywords:** pedagogical instruction, curriculum, generalists, in-service teacher, professional development

## Abstract

Physical education (PE) is beneficial for the development of elementary school-age children through its promotion of different educational learning outcomes, which in turn affects the long-term development of physically active lifestyles. In many countries, PE is taught by classroom teachers (CTs), who are thought to be in a unique position to positively impact students’ learning. While a substantial body of studies examines the challenges that CTs encounter when teaching PE, less research has been directed towards gaining a comprehensive understanding of how CTs visualize PE and, in turn, how to promote various types of PE. The purpose of this study, therefore, was to explore elementary school CTs’ beliefs about and practices in PE in South Korea. To achieve the research goal, a semi-structured face-to face interview with six CTs was performed to collect the qualitative data source, using the ground theory as an analysis method. Three themes emerged from these interviews concerning the CTs’ beliefs in PE: (a) the importance of understanding students’ characteristics, (b) the importance of centering internal perceptions in PE method, and (c) the importance of meaningful experiences. The results also identified two themes for teaching practices in PE that corresponded to CTs’ beliefs: (a) crafting personalized instructional methods and (b) connecting PE experiences. This study lends important insights to future practices and research recommendations for CTs’ PE teaching and teacher education programs.

## 1. Introduction

Over the last few decades, elementary school physical education (PE) has garnered attention across a range of socio-cultural, political, professional, and academic discourses around the world (e.g., [1,2,3]). Much of this attention has largely been due to a growing perception that formative PE experiences have the unique opportunity to address various common concerns about children’s development of motor competence [4] and social-emotional learning [5], which in turn affects the long-term development of physically active lifestyles [6]. In addition, PE experiences provide opportunities for children to engage in moderate-to-vigorous physical activity, which has been proven to critically impact the healthy development and well-being of school-aged youth [7,8]. While research indicates that PE programs have the potential to benefit students, the programs must be appropriately designed and intentionally delivered in order to be successful—what teachers do in the classroom and how they interact with their students has a significant impact on student achievement and development [9].

In many countries, especially in Asia, elementary PE is primarily taught by classroom teachers (CTs: generalist teachers who teach every subject) [3]. CTs are assigned to teaching PE because of their relative experience in building positive relationships with students and their capacity to understand individual’s learning needs [10]. Although the importance of PE for elementary-aged children has been well-established in the literature [11], scholars have reported that PE programs, when delivered by CTs, lack quality, especially when it comes to cultivating meaningful experiences and achieving student learning outcomes [12]. Part of this issue may be related to inadequate teacher training in PE [13]. Given the significance of conquering CTs’ lack of preparedness to teach PE, it is vital to consider their values/beliefs on the subject and the influence these have on changing their PE pedagogy.

In the research on teaching effectiveness, teacher belief has historically been considered a critical component of quality teaching [14,15,16]. This research has contended that teachers’ beliefs—about their students, instructional methods, and subject matter—are an integral aspect of the teaching process [17] and are assumed to have a significant influence on teachers’ instructional decisions and practices: behaviors that ultimately impact students’ learning and positive development [15,18]. It should be noted, however, that the research on this topic indicates that teachers’ beliefs can compete with each other; sometimes, act as contradictory discourses that (in)form; and at times, impede effective practice. These contradictions may be due to personal experiences and context factors (e.g., school environment and administrators’ support) [19].

### 1.1. Review of Literature

#### 1.1.1. Defining Beliefs

Beliefs are assumptions held by individuals about the world and oneself based either on their own experiences or on external authorities [20]. Beliefs can be also conceptualized as an “individual’s judgement of the truth or falsity of a proposition” [15]. Beliefs are characterized by their “unboundedness” with variable, uncertain linkages to events, situations, and knowledge systems [21]. Nespor [22] argued that beliefs operate separately from the cognitive activity related to creating knowledge, while Bandura [23] stated that beliefs, more than truth, guide our goals, emotions, decisions, actions, and reactions. Although there is some degree of elasticity to beliefs, such that they can change with time and/or experience, beliefs are more or less consistent within the individual [24].

#### 1.1.2. Educational Research on Teachers’ Beliefs

Beliefs are an important concept in understanding teachers’ decision-making processes, teaching practices, and their participation in teacher education programs [15]. The literature depicts teachers’ beliefs as a “messy construct” due to inconsistencies in defining and operationalizing terms [25]. For instance, Richards [16] noted that teachers’ beliefs refer to “the information, attitudes, values, expectations, theories, and assumptions about teaching and learning that teachers build up over time and bring with them to the classroom.” Pajares [15] also pointed out that educational beliefs affect one’s confidence in a variety of ways, including the ability to influence student performance, knowledge, perceptions, and personal feelings; the ability to perform specific tasks; and the perceptions of ability in specific subject matters or disciplines. Fang [26] provided a more practical, concrete definition of teachers’ beliefs: the general knowledge of a teacher’s students and the lesson contents that influence a teacher’s decision making as well as their classroom practices. In spite of different perspectives on the definition of teachers’ beliefs, the term “teachers’ educational beliefs” has been generally used to point to what teachers value in teaching practices [15]. In other words, teachers’ beliefs play a significant role when choosing what content to teach and organizing the knowledge relevant to the task [27].

The importance of understanding and articulating teachers’ beliefs is evidenced by decades of continued theoretical and practical research (e.g., [14,15,16,27,28]). In the field of PE, research on teachers’ beliefs frequently has been focused on concepts related to pre-service teacher preparation [14] and intended curricular outcomes [29]. Morgan and Hansen [30] found that CTs’ beliefs about the importance and value of their work was closely related to their professional practices across subject matters. Similarly, Wilkins [31] found that for 481 elementary school teachers in the United States, beliefs about the effectiveness of inquiry were the strongest direct predictor of inquiry-based instructional practices. In Europe, Tsangaridou [32] interviewed preservice CTs before a 13-week student teaching experience, observed six periods of PE lessons per teacher, and analyzed relevant documents (i.e., lesson plans and reflective journals). Based on these data, the author concluded that the student teachers’ articulated beliefs about PE were reflected in their practices. In Hong Kong, Qi and Ha [33] illustrated the importance of subjective beliefs and perceived behavior control on the decisions of teachers to teach students in inclusive PE settings based on a qualitative analysis. Although these studies occurred in different geographical environments, all teachers’ beliefs were identified as a predictor of both classroom goal structures [34] and instructional practices [35].

#### 1.1.3. Teachers’ Beliefs and Practices

As Thompson [36] argued, “The relationship between beliefs and practices is a dialectic, not a simple cause-and-effect relationship” (p. 140). Although, as demonstrated in the research cited above, teachers’ beliefs are related to practice and beliefs about practice, they are not simply able to be mapped onto another. In other words, beliefs and practices influence one another, and the strength of this relationship, as well as the type of beliefs, may vary across individuals and contexts [16,36]. Even though teachers implement the same curriculum, teachers’ practices in class differ based on their educational beliefs [37]. However, researchers who explore teaching have often overlooked the degree to which beliefs impact teachers’ practices. Moreover, while many studies on education (e.g., [38]) and PE (e.g., [11,32,39,40]) have shown consistent alignment between teachers’ beliefs and their actions, other research has demonstrated inconsistencies between these concepts [41,42]. Kulinna et al. [43] investigated the alignment between in-service teachers’ beliefs about physical fitness and the content they taught. While the researchers expected that their participants’ beliefs in the importance of physical fitness would correlate with higher levels of moderate-to-vigorous physical activity in their classes, they found no relationship between the two. Although prior research indicates that the relationship between teacher’s practices and beliefs is important, knowledge is limited on the nature of the intersection between the two—hence, more research is warranted. Despite the lack of consensus about how a teacher’s beliefs and practices influence one another, CTs are now more likely than ever to assume the responsibility for providing PE lessons. Exploration of these concepts and their interaction is vital, as they can be essential to developing quality teacher education programs.

### 1.2. PE in the South Korea Elementary School System

In South Korea’s public elementary education system, schools provide 6 years of education for children aged 7 to 13, also known as primary education (Grades 1–6). Among all elementary school teachers, there is a significantly higher number of female teachers (77.8%) than male teachers [44]. Generally, PE is a required subject and is taught by either a CT or a PE specialist. However, predominantly more PE classes are taught by CTs rather than specialists. CTs’ curriculum consists of Korean, English, mathematics, science, social studies, life studies, moral education, arts, music, and PE.

According to the national curriculum guidelines from the Korean Ministry of Education [45], the primary aim of PE is “To teach a holistic education by acquiring the competencies of the PE subject through internalization and practice of physical activity values.” Specifically, the national PE curriculum is organized in five components: (a) health domain (e.g., stretching, bicycling, and push-ups); (b) challenge domain (e.g., gymnastics, track and field, and martial arts); (c) competition domain (e.g., soccer, basketball, and volleyball); (d) expressive domain (e.g., traditional dance and creative dance); and (e) safety domain (e.g., CPR training) [45]. Specific physical activities and/or sports can be selected by CTs who teach PE if the activities align with the national curriculum and are in consideration of the educational conditions of the school (i.e., facilities, environment, resources, and administrators). PE lessons are typically 40 min at least twice per week, and a total of 90 mandatory hours per year is allocated for PE in public elementary schools. PE also plays a primary role in extracurricular activities (i.e., before- and after-school programs), sport club events, and sport festivals in elementary schools. However, due to inadequate preparation that often arises from the need to teach every subject, teachers lack necessary in-depth subject-specific practices, knowledge, and learning opportunities for teaching PE.

### 1.3. Research Framework and Purpose

Tsangaridou [46] demonstrated, in accordance with Fuller’s [47] framework, that educators in PE hold strong feelings about ten categories of teaching issues: (a) beliefs of recruits and prospective teachers about the purposes of PE, (b) beliefs about learning to teach and teaching experiences, (c) beliefs about effective teaching, (d) beliefs on teaching PE, (e) beliefs about learners and learning, (f) relationship of beliefs to teaching practices, (g) beliefs about subject matter, (h) beliefs about self and the teaching role, (i) beliefs about the nature of their work, and (j) teacher concern. Derived from the aforementioned theoretical framework, the researchers focused on two relevant categories for the study: beliefs about subject matter and teachers’ beliefs on teaching PE. We expected that understanding CTs’ perceptions of PE as a subject would help to explore the relationship between beliefs and practices in PE as well as their approaches. Specifically, this research explores “teachers’ practices in teaching PE,” which we defined as any action that is part of the teaching process and strategies such as planning, decision making, instructional approaches, and relationship building with students. Overall, the purpose of the study was to investigate South Korean elementary school CTs’ beliefs and their practices and experiences about teaching PE. The research questions that guided the study were as follows:

(a) What are CTs’ beliefs about teaching PE?
(b) To what extent are CTs’ teaching practices in PE based on their beliefs?

## 2. Methods

### 2.1. Study Context and Participants

Participants were six female elementary school teachers who voluntarily agreed to participate in the study, all from three different metropolitan cities in South Korea. In South Korea’s primary education context, a CT (generalist) or a specialist teaches PE. However, in most elementary schools, CTs, rather than specialists, have the responsibility of teaching PE. The participants in the study were elementary school CTs, specifically those with enough experience in the field of teaching PE. The researchers selected participants with a purposeful sampling method [48]. The researchers specifically targeted and approached CTs who met the following criteria: (a) teaching experience of 10 years or more; (b) instruction in various activities for the professional development of CTs (e.g., instructing workshops/training, making useful teaching materials, and mentoring activities); (c) high evaluation for excellent PE teaching practice; (d) positive teaching research activity (i.e., degree and/or publications); and (e) rapport with the investigating establishment. Pseudonyms of the six participants and their demographic backgrounds are indicated in Table 1.

### 2.2. Data Collection

Interviewing is a powerful way to gain insight into the educational and social phenomena experienced by individuals in educational contexts [49]. The research method for this study involved a descriptive-qualitative approach using in-depth, semi-structured interviews, which are typically utilized when interviewing an individual [50]. The aim of the in-depth interview method was to identify and explore implications about the participants’ beliefs and concrete teaching practices based on their various teaching experiences [48]. Trained and experienced qualitative researchers conducted interviews at the school where participants taught PE classes. This allowed the researchers to observe the teaching methods and the teaching materials/equipment used during class.

A total of two interviews, each lasting between 34 and 47 min, were conducted with each participant. The first interview questions focused on three categories: (a) what the teachers believe about teaching PE (i.e., goals, priorities, teaching methods, values of PE, and teacher’s ability/role); (b) what the teachers observe in their classes (i.e., what actually happens in PE classes); and (c) what the teacher plans for and uses in the PE classes to facilitate their teaching practices (i.e., what they think should be happening). Based on these open-ended questions, the researchers gained a generic understanding of CTs’ beliefs and perspectives about teaching experiences in PE lessons. All first interviews were reviewed, and the researchers developed tentative routes of exploration for the second interview. The second interviews enhanced the richness of the data collection as participants more willingly shared intimate details of their lived experiences. Participants were questioned about various types of instructional approaches and how they might maximize physical activity opportunities in and outside of PE lessons. Specifically, the relationship between CTs’ values in PE and their pedagogical practices were addressed in the second interview.

### 2.3. Data Translation

This study used the cross-cultural translation technique adapted by Banville et al. [51]. The two researchers who were fluent in both English and Korean independently translated the original Korean version of interview answers into English. Next, the researchers compared their versions of the translations and discussed any differences until they arrived at an agreement. Translated data were then revised as deemed necessary for proper vocabulary and syntax. Lastly, the original Korean recordings from this process were sent to an external reviewer for ensuring the meaning of the English and the original items were the same.

### 2.4. Data Analysis

The data generated were initially analyzed using the procedures and techniques of an inductive and a constant comparative methodology [52,53]. Specifically, for this study, a grounded theory approach was utilized, as it was the researchers’ aim to “discover relevant categories and the relationships among them, to put together categories in new, rather than standard ways”[54] (p. 49). This approach consists of two main operations: (1) breaking down the data into meaningful units and (2) grouping units with similar meanings into broader categories [55].

First, all the in-depth interview data of participants were audio recorded and transcribed in Korean (i.e., initial transcription). Next, the interviews were translated into English, independently coded by the lead author, and checked by the second author using an open-coding approach, thereby creating an initial code list from the data. Through the coding process, the researchers primarily responsible for the analysis developed theoretical sensitivity (sensitivity is attained through stopping and thinking anew, by considering multiple vantage points, comparing, and building on ideas and themes) [56] during the analytical process, increasing insight and understanding about the participants’ beliefs and teaching practices through interaction with the data [52]. After completing the open-coding process, axial coding was conducted to discern relationships between the codes. During axial coding, “a process whereby data are put back together in new ways after open coding by making comparisons”[54] (p. 96), a constant comparative method was used to cluster the participants cases, which were then compared to identify similarities and differences. All data segments that related to a specific code were sorted through a procedure referred to as “creating categories.” The categories produced in this process became more detailed as the key themes became established through repetitive and comparative examination of the text [53]. The data were continually checked against reviewers’ interpretations until all were in agreement that they had grasped the common themes [52]. Throughout the analysis, the selective coding occurred. This process involved selecting the core category, systematically relating it to other categories, and validating those relationships and filling in categories that needed further refinement. The results produced through selected coding were guided by the authors’ knowledge of the technical literature.

### 2.5. Trustworthiness

To ensure the soundness of the data collection and analysis, three strategies, guided by Lincoln and Guba [57], and Patton [48] were implemented. First, triangulation was conducted to ensure that the findings were accurate using multiple data sources, including those from interview transcripts. The same data resource was independently analyzed by two researchers, and their findings were compared. Email conversations with participants were also used to check the transcribed data (internal validation). This was carried out to avoid ambiguous interpretations, doubts, and/or different responses, which could affect the method’s accuracy, data collection, and in-depth analysis procedure. Second, peer de-briefers were used to examine developing interpretations and to challenge the researchers to support their interpretations with data. They deemed the interpretations of the data to be accurate and representative. Finally, member checking was used to reduce the impact of subjective bias: the participants were asked to read the interpretations of the data and to correct any inaccurate information or interpretations.

## 3. Results and Discussion

This section explores CTs’ beliefs and practices in teaching PE as generalists. The findings are divided into two sections: (a) CTs’ beliefs about teaching PE and (b) CTs’ practices for teaching PE. CTs have a variety of backgrounds, but based on their unique previous teaching experiences, they have subjective beliefs about and unique teaching approaches for PE. The themes are summarized and concurrently discussed in this section, with associated subthemes embedded in the text and supported with direct quotes extracted from the CTs’ interview transcripts.

### 3.1. CTs’ Beliefs about Teaching PE

#### 3.1.1. Importance of Understanding Students’ Characteristics

CTs believe that understanding the different developmental levels of their students, both physically and emotionally, is critical for successful student engagement in PE lessons. In relation to these lessons, the teachers commonly addressed class climate and student emotion.

##### Consideration of Class Climates

Most CTs noted that they, with their students, discuss in advance the factors that may lead to conflict between students in PE class. Dasul, in particular, addressed the use of precorrection (clearly stating behavior expectations before students have an opportunity to misbehave):

I believe that conflict always affects the mood of students. Before participating in a catch-and-play activity, we should talk about our emotions and how to manage them so as not to be upset during the lessons… If I give precautions to children in advance like this, I think it works better…it makes students more focused on the class and allows them to engage in activities comfortably.

(First Interview)

Similarly, in discussing the effect of PE curricula and programs on class climate, Cheolsoo noted.

I believe that the students’ feelings about PE and the climate of their classes affect PE classes… When we are looking at the PE curricula, there are so many different game activities. However, most of those classes are simply divided into teams, and as a result, one team wins, and the other loses. When these activities are repeated, a competitive climate naturally grows in classes, and students compare with each other... [as a result], some students feel afraid of PE lessons, and their dissatisfaction grows. I don’t think it’s desirabl.

(First Interview)

Both CTs’ descriptions of the importance of considering class climate demonstrate how many CTs experience contextual factors that influence students’ engagement during PE lessons. Specifically, one of the CTs within this category reported that promoting a competitive climate may discourage students from actively participating in PE lessons. She appeared to be concerned that such teaching approaches and programs increase withdrawal among students, particularly low-skilled students; this concern aligns with previous research findings [58,59]. Furthermore, CTs highlighted that teachers should promote appropriate student behavior in PE lessons by responding to it with recognition and affirmation. In a study by Moon et al. [60], proactive management strategies were revealed to be negatively associated with disruptive (i.e., interference with classroom activities) and aggressive student behaviors (i.e., aggression toward teachers or peers), while reactive strategies were shown to be positively associated with these student behaviors. Thus, effective instructional management and proactive management approaches are mutually beneficial and reinforce students’ learning in PE.

##### Effects of Emotions on Engagement

Most CTs believe that it is crucial to understand and guide students’ emotions during PE. In other words, CTs should consider the attitudes of students toward their activities and relationships in various situations, both inside and outside of PE classes. As Ahn-wook expressed that this belief is thought to be associated with the creation of an environment in which students can focus on PE:

It is important to understand students’ minds and attitudes because PE classes are required, and students need to actively participate. For example, when a student is not feeling well or has emotional distress, the student resists participating in activities. As such, the teacher needs to understand and help these students.

(First Interview)

Boram builds on the idea of monitoring students’ emotions, emphasizing the importance of checking student’s feelings even before instruction:

Sometimes the teacher must understand students’ emotions before instructing them to be active with their bodies... At the beginning of each lesson, it is very important for a teacher to understand what attitude and feelings [students] have, because the individual characteristics of the students will have an overall impact on each PE class.

(First Interview)

Interestingly, Boram and most of the interviewed CTs’ described emotion as a critical aspect of motivation, behavior, and commitment in PE. They assert that emotional engagement entails affective reactions in the classroom. These findings align with previous studies, where CTs believed that a positive climate in PE plays a significant role in students’ motivation [58,61]. Wright [62] underscores that the goal of elementary PE is to provide children with a variety of positive experiences that help them maintain the essential intrinsic value of enjoyment, which in turn encourages them to continue participating in physical activity [9,63]. Since elementary school-age children are at the period during which the capacity to acquire personal autonomy develops, it is critical for CTs to teach children to recognize and understand their own emotions as well as the emotions of others.

The above findings imply that motivational climates which promote positive emotions have been shown to have a significant impact on students’ PE participatory experiences in a variety of settings [64]. Furthermore, teachers play a pivotal role in creating a safe and supportive environment that encourages student engagement in PE lessons. As a result, CTs should recognize and identify students’ needs in PE instruction based on their understanding of their students’ emotions and various other developmental characteristics.

#### 3.1.2. Importance of Centering Internal Perceptions in PE Method

Another recurring motif among the interviewed CTs was that it is insufficient for teachers to provide merely skill-oriented training opportunities to the students in PE classes. They emphasize the significance of shifting PE instruction away from physical goals (i.e., performance-centered approaches, developing sports skills) and toward internal perceptions as a learning outcome. The teachers shared beliefs that there are several approaches to achieving this viewpoint.

##### Moving beyond Skill-Centered Approaches

CTs believe that students should be free from a traditional skill-centered approach to PE that focuses only on developing individual skills. That is, acquiring sport or motor skills is only one of the outcomes that can be gained through PE classes. On this topic, Hyejin describes how the teachers changed their perspectives about PE outcomes:

Traditionally, I think many elementary school teachers have a strong awareness of the importance of the skills-centered activities… Many teachers around me said that learning good skills is a priority in PE classes; if students learn skills well and teachers did good work, the children are getting good PE classes… But, I don’t think so…developing functional skills is one of the important parts of PE classes.

(First Interview)

Additionally, most of the CTs believe that it is necessary to escape from the traditional PE culture centered on iterative mastery of performance-centered, game-oriented, competitive, and skill-drill related tasks. Dasul mentioned:

It seems that the skill and performance-based lessons can negatively impact students’ sense of competition. I have often watched students blaming and criticizing each other. I believe we should teach classes that deviate from this culture. PE is not for fighting and criticizing each other. Also, it is wrong to compete and discriminate according to the students’ ability… I think that teachers should turn to a culture within PE classes that respects each other’s diversity and enables everyone to participate happily together.

(First Interview)

Eunhee offered some specific examples of how performance-oriented instruction may be harmful:

When students play a basketball game during PE, the student with the strongest physical skills has the advantage. In this way, students with good motor skills will have an enjoyable experience, but relatively low-skilled students will be in the opposite situation. They get marginalized from participation. I believe this type of activity is meaningless to students and diminishes their motivation to participate.

(Second Interview)

Cheolsoo also addressed a particular example:

I think it is a very limited view to focus only on improving students’ fitness. Participation in physical activity allows students to learn not only skills but also various virtues such as cooperation, care, communication and sportsmanship. I think these are another important learning outcome of PE classes, and I think that the performance-centered approach is not enough to be a good class.

(First Interview)

The CTs explained that traditional skill-oriented lessons could both unintentionally benefit students who already exhibit high performance and could negatively affect other students’ motivation and engagement in PE lessons. They emphasized that students should develop positive values and different outcomes such as social development and healthy physical development through PE classes. DeCorby et al. [65] found that CTs perceived PE as vital to the development of the whole child, particularly in terms of physical and social development, which is consistent with the current findings.

In addition, the majority of the CTs expressed concern about reinforcing skill-oriented instruction regardless of the lesson content. This instruction focuses on improving the ability to outperform another participant, on-field performance—teachers in this environment pay more attention to the best players. These tend to emphasize intense, highly competitive practices and games, with the team’s primary focus being on winning the game. In line with the results, previous research has identified that it causes students to experience anxiety, fear, pressure, and lack of interest in PE [66]. Thus, CTs should adopt strategic techniques to transcend beyond traditional skill-centered approaches in PE classes in order to accomplish a variety of learning outcomes (e.g., social and affective domain) with children from different backgrounds.

##### Intentionally Encouraging Internal Values

CTs contended that students are learning and practicing purposeful values in PE classes. Further, the CTs’ beliefs influence the planning and organizing of classes that help students achieve their intentional educational values and goals. Concerning the internal values included in PE, Boram stated, “Because various situations arise in PE lessons, it is very suitable for teaching a variety of values such as consideration of one another, following the rules, fair-play, good sportsmanship, and doing one’s best.” Hyejin intentionally designed the contents of PE lessons based on her teaching beliefs. She described her belief and reasoning as follows:

Giving students physical activity opportunities and teaching various movements are the main purpose of PE classes. But, beyond this, I believe the teacher could put intentional thoughts behind the physical activity into the lessons. I think this can provide children with holistic learning opportunities as well as physical activity.

(First Interview)

In relation, Quay and Peters [67] addressed that PE learning to design and play games in a social-emotional setting may produce opportunities for PA engagement outside of PE lessons. Thus, incorporating internal values into PE may facilitate CTs moving beyond traditional skill-centered PE, which tends to emphasize individual success at the expense of collaboration. In concurrence with this perspective, Ahn-wook also shared her unique perspective on the significance of acquisition of internal values in PE classes:

Based on the national PE curriculum, I believe the teacher should design the lesson with the goal of maximizing students’ learning… Have students wear team vests with important values printed on them when doing group activities: honesty, respect, sportsmanship, communication, these things. I believe learning these values are vital for students’ development.

(Second Interview)

The CTs’ beliefs included expanding intentional PE instruction to include internal values and attitudes for students’ learning. The findings indicate that understanding the role of CTs in teaching values (e.g., respect, fair play, and teamwork) in PE lessons is critical for designing the lessons. Previous research demonstrates that the values, character, and conceptions of the teacher all have a significant impact on the formation of values among his or her students in sports and PE [68]. However, Jones [69] states that teachers understand values as something transmitted automatically and not as something that is consciously learned—these teachers do not realize the importance of planning their lessons to explicitly encourage learning values. Jacobs et al. [70] also illustrated that teachers believe that PE could contribute to the acquisition of values, but their methods are rarely deliberately planned. Studies are warranted to investigate the effects of intentionally planned CT teaching approaches for communicating values in student learning. Overall, these CTs advocate that the value of PE is in helping students improve educational learning outcomes, which should be developed through their instruction.

#### 3.1.3. Importance of Meaningful Experiences

Many of the CTs noted the importance of providing meaningful experiences to students in PE classes. Meaningful PE is a pedagogical approach to PE instruction that is designed with the aim of helping teachers explicitly prioritize meaningful experiences for students [12]. The CTs strongly believed that it is important not only for students to learn sports skills but also for them to enjoy participating in PE lessons. Specifically, this belief relates to students’ successful achievement experiences, which gives students motivation to engage in physical activity in the long term. Ahn-wook stated the necessity to constantly strive to create this type of context for students:

Significant engagement of students in PE is helping to eliminate fear when they try the exercises they already learned again. Teachers have to instruct students to experience their own successes and enjoy them. Eventually, meaningful experiences build up, making them self-confident and constantly engaged in PE classes.

(First Interview)

Boram spoke to this, using a specific teaching example about a meaningful PE class:

Even if students participate in the T-Ball game once, I think the physical activity experiences that they enjoyed when they were young… will have a positive effect on their quality of life in the future. An hour of PE class may not mean much to the teacher. However, for students, I believe that it can help them develop a physically active lifestyle because they are able to have meaningful experiences through enjoyable PE classes.

(First Interview)

Cheolsoo emphasized the importance of the teacher’s role in providing meaningful PE experiences and provided additional support for pursuing more widely meaningful PE classes when she explained the long-term relationship between engagement and quality of life:

It is important for students to encourage each other rather than compete in PE class and to make them feel more comfortable and confident about participating in various sports activities … students have a desire to participate in lifelong physical activity through meaningful PE experience, which ultimately affects the quality of life.

(First Interview)

Finally, Eunhee emphasizes how the accumulation of meaningful experiences in PE contexts is important from a long-term perspective, which is one of the essential goals in PE:

I think that meaningful PE classes for each student can grow up step-by-step like this: a little better than before, tomorrow’s time is better than this time today. I believe this will eventually have an effect on students’ long-term participation in physical activity… This shows why PE matters for children.

(Second Interview)

All in all, the majority of CTs stated that meaningful PE experiences made students enjoy PE more and produced long-lasting effects. Thus, these meaningful PE experiences should be nurtured in early developmental stages [2]. More specifically, promoting meaningful PE experiences in this period would positively impact the likelihood of students having long-term healthy and active lifestyles [6]. This belief helped CTs be more intentional in their pedagogy and in their prioritization of meaningfulness in their PE classes, which is in line with the research. Beni et al. [12] demonstrated that CTs’ implementation of meaningful PE experiences was inextricably linked to their positive beliefs about the approach, in terms of their perceptions of beneficial student learning outcomes and in drawing connections between approaches and beliefs in teaching PE. Essentially, as will be demonstrated below, the CTs’ various beliefs about teaching PE are important because they filter down to and inform their practices and methodological approaches for supporting students learning in alignment with the national PE curriculum.

### 3.2. CTs’ Practices for Teaching PE

As indicated above, CTs had various teaching practice experiences based on their different beliefs about PE and what it should accomplish. This section explores how teaching practices are implemented in concert with the CTs’ beliefs.

#### 3.2.1. Crafting Personalized Instructional Methods

CTs use their own interests and pedagogical knowledge to design novel teaching methods as a strategy to implement PE lessons. Boram stated, “It is important for the teacher to design a unique lesson plan and to present interesting activities to the students in order to achieve the goal of student learning.” There are, then, multiple ways to personalize instructional practices.

##### Flexibility of Pedagogy

CTs’ practices were underpinned by directives laid out in the national PE curriculum. They also recognized that the important aspect of PE is not only to teach one single lesson but also to create a long-term plan over a period of a month or semester, which gives students enough time to learn the relevant skills. For instance, Dasul stated, “I think it is important to expand and expose the elements related to the development of health-related fitness components frequently throughout school days.” Hyejin’s explanation also demonstrates this practice:

The 5th grade curriculum includes cultivating healthy related-fitness. So, I planned a class that teaches students how to exercise to improve cardiorespiratory endurance, strength, and agility. For example, to build strength, you have to run stairs, and to develop agility, you have to run 50-m or long jump. However, when I analyzed the PE curriculum in detail, I found that the students not only need to do these exercises, but they also need to cultivate a willingness to pursue a physically active lifestyle and to realize the importance of persistence in cultivating health and physical strength. So, I designed my own class using the cooperative learning method. I provided a certain period of time for each group to make a plan and take videos of their practice activities while keeping track of their records and writing an exercise journal.

(Second Interview)

Hyejin and other CTs pointed out that adaptation to the level of students is a more effective teaching approach than following the national curriculum word for word. Previous research demonstrates that teachers are encouraged to actively engage in national PE curriculum flexibility to become creative and adaptable professionals who can employ the ideas that arise when students from different backgrounds are immersed in their own learning process [71]. Thus, the interviewed CTs focused more on the content, its development, and how best to modify and teach content in their curriculum in order to maximize PE’s potential benefits to students.

To this end, the use of CTs’ specific knowledge and interests allowed for approaches that could help teach students with a wide range of skill levels and degrees of experience. Cheolsoo has developed task progressions in various ways based on student characteristics to effectively present content in PE. She stated:

When teaching a specific activity, due to students’ developmental stage, I think elementary school teachers have to present tasks in very subdivided progressions. Even when divided into beginner, intermediate, and advanced levels…the movements must be segregated and presented progressively so that the children will recognize the skills and then their bodies will follow how to do it.

(Second Interview)

As Cheolsoo and most of the CTs stated, for elementary school, teachers should provide students with “modified game activities” rather than “an entire sport game” so that students can easily understand the game and maintain motivation to participate in activities. They highlight how modifying the type of game activity and instructions should occur in order to meet the developmental needs of their students. In accord with these findings, Ward and Ayvazo [72] described that the quality of content representations in PE (a) are sufficiently detailed to convey understanding to the students, (b) are appropriate for the student’s developmental level, and (c) make connections to existing performance and/or knowledge of the students. Both researchers and practitioners, then, articulate the value of contextually modifying activities.

Another practice that teachers and researchers advocate for is re-organizable and adaptable content for task progression for particular students and classrooms. To this point, Ahn-wook was able to identify the shortcomings of and make adjustments to her pedagogical approach in accordance with her students’ abilities and adapt the task progression for students’ different levels in lesson contexts:

One day, during gymnastics, one of the students asked me, “Why are we learning to roll forward?” It was really difficult to answer at that time. A regular answer is maybe, “It’s a basic skill to do various movements well, and if you do this well, you can do other exercises well.” But, maybe this explanation makes it difficult for students to understand the class goals. So, I gave them specific situations that require them to use the “roll forward skill” while I explained why we should learn this. For instance, if you fall while talking with a friend, but you roll forward and get up, you can prevent injury. Students could understand the purpose of content much better.

(Second Interview)

In the above example, Ahn-wook was able to adapt to the particular contextual needs of her students and to adjust the task progression accordingly. CTs have to adapt to and prepare for the environmental factors that may have an impact on PE teaching. Cheolsoo emphasized a flexible pedagogical approach that accounts for these factors:

When the weather is bad or when I can’t teach PE classes in the gym or outside of class due to school events, I teach the classes using content related videos as a backup plan. Through videos, I can introduce students to the Olympic Games or to different countries’ sports cultures. Watching these videos also teaches the attitudes, dispositions, and important values.

(Second Interview)

A strategic method similar to Cheolsoo’s practice is required to ensure the educational objectives and values of PE are communicated to and taken up by the students in any environment and situation. Overall, the CTs advocated for a flexible and responsive pedagogical approach to the particulars both of the students and of the situations that affect students’ ability to achieve positive physical and emotional outcomes through PE lessons. CTs need to have a variety of flexible teaching approaches (e.g., changing the schedule or using metaphors to help students understand the tasks) that are derived from previous teaching experiences, content knowledge, and their beliefs in PE.

##### Providing Various Options

CTs expressed that a good PE environment needs to give students individual attention. In other words, they try to avoid teaching methods that unilaterally lead the students to the learning objective set by the teacher. Hyejin shared her teaching practices which illustrate this principle of individuation:

When I taught frisbee lessons to my 3rd graders, they had a hard time throwing and catching the frisbee during the activities. [The frisbee] flies too fast and it was small. So, I used different equipment in the lesson… The change in equipment was effective because I kept the rules of the game and just replaced it with a light and big ball instead. It was a lot more fun for the students to participate in class.

(Second Interview)

CTs prefer to create inclusive PE lessons to respect the diversity of students’ experiences. According to Dasul, she allowed students to organize the contents of a PE class in order to provide participants with engaging experiences:

In the case of transforming games, there are no rules that must be followed, so I work with the students to create the rules for the games. It became a much more effective activity as we talked about the modified rules that we must follow before the game and then discussed the pros and cons after the game.

(First Interview)

Similarly, Boram designed various activities for the students who have different skills and backgrounds to achieve the goal of successful student learning. She described:

When I taught baseball lessons, I gave students the opportunity to take on a variety of roles. It’s not just attacking and defending, but also acting as a referee or a scorer, and some of the students even took on the role of reporting the game.

(Second Interview)

The CT’s practices of using different options may provide more opportunities to develop each individual students’ PE learning outcomes effectively. The testimonies of the CTs cited above demonstrate how each teacher tailored their lessons according to their own beliefs, ensuring students’ internal values and needs through meaningful PE lessons. Since the modified PE lessons and/or curricula present unique opportunities for students to choose the activities in which they participate, they can be able to describe their feelings of enjoyment that they derive from participation. This is in line with the research, which states that activities that spark the interest of students increase the likelihood of their participation, while activities that are not perceived as interesting decrease this likelihood [73]. In light of these results, CTs may aim to plan for a variety of experiences that allow their students to access features of meaningfulness over time (e.g., social interaction, enjoyment, and personal characteristics).

#### 3.2.2. Connecting PE Experiences

CTs provided students with opportunities directly connected to in-class activities that they could practice beyond class hours to achieve PE goals. Hyejin noted that she “never thinks of PE time as its own activity,” treating PE instead as an opportunity to create *life* habits. CTs articulated different ways to maximize this learning potential through technology and communication in settings that may support the learning of PE. For instance, Dasul used technology (via flip learning) related to tasks before and during the class to help students understand activities and to improve academic learning time in PE. Other CTs used the approach of communicating with the students to encourage their constant and voluntary engagement in active lifestyles. Some CTs, for example, recorded scenes of lesson activities and shared the class scene with students and parents by using either an app or the classroom website. Eunhee addressed her teaching experience with this practice as follows:

I recorded a video of gymnastics lessons that were especially difficult for the students and shared it via the app. Students can see where they did not succeed at first, but also see their progress. They can check it at home, so they can watch the video with their parents.

(First Interview)

Through video sharing of the lessons, students were able to discover their self-development and stimulate interest in PE. Parents also had the opportunity to identify students’ skill developments and actively interact with the teacher. Ahn-wook mentioned the following based on her experience teaching a PE class:

When students have a hard time learning skills, take a video and show them, so the kids understand it much better. And if you share the video, go home and watch it yourselves. Students are more pleased as they discover their progress. I think it’s much more motivating. And if I share the video of the students using the shared platform, they can watch it with their family. Students are happy as they observe their progress with family. These things seem to motivate them to participate in PE class actively, which encourages them to pursue physically active lifestyles in and out of school.

(Second Interview)

These results support that the practice of fostering a culture of participation can be better achieved by providing students opportunities for PE activities both at school and at home [74]. The majority of CTs demonstrated various strategies for extending PE activities beyond the classroom, including opportunities and activities in which parents can become involved. Bandura [75] stated that enlisting parental involvement in school activities positively contributes to students’ ability to transfer skills to activities they may be able to carry out at home. This practice reflects the CTs’ belief that it is critical for students to enjoy PE with a positive attitude, which supports the development of meaningful experiences and establishment of long-term goals, especially the goal of creating lifetime physical activity participants.

## 4. Conclusions

While there has been a significant body of research working to explore the issues CTs face in teaching PE [51], less research has been conducted that works to understand how CTs visualize PE and how to promote different types of PE. Indeed, only a small amount of research has been performed to gain a comprehensive understanding of CTs’ PE beliefs and practices and how they inform one another; this study has attempted to address this gap in the research. The purpose of this study, therefore, was to investigate South Korean CTs’ beliefs, as generalists, on teaching PE lessons. Another goal was to investigate to what degree their practices in teaching are based on their beliefs about PE. A thorough analysis of the qualitative data was used to strengthen our understanding of this phenomenon in order to achieve research goals. Regardless of the participants’ various beliefs, they all emphasized the importance of teaching internal PE values and providing meaningful PE experiences to students. The results also reported that their beliefs in PE influenced their decision to use personalized instruction and to maximize engagement opportunities. The findings of this study can help to fill a gap in the existing body of research by providing not only the beliefs and practices themselves but also the connection between beliefs and practices, particularly those related to PE.

There are some limitations of the current study. One limitation of this study was the teaching experiences of the participating CTs. All of the CTs had many years of teaching experience, so there was a lack of novice CTs who may have different belief systems, perspectives, and teaching approaches in PE. Additionally, the relatively small number of teacher interviews, and the fact that all of the participants were female may impact the generalizability of the findings to all of South Korea’s teachers or generalists. Nevertheless, this study lends important insights to future practices and research recommendations for CTs’ PE teaching. Specifically, the practical implications of the study are as follows: (a) in order to increase students’ motivation and engagement, CTs need to consider their social–emotional climate and to use proactive instruction strategies in PE classes; (b) in order to provide meaningful and successful experiences to students, CTs need to incorporate more adaptable pedagogical strategies in a variety of contexts (e.g., giving students different options to create inclusive learning environments in- and out- of the classroom).

Future research should be conducted to investigate the beliefs and practices of CTs from a wider range of backgrounds. Given the findings from the teachers’ self-reports, additional investigations should employ methodology that allows for quantitative analysis and systematic observations to compare CTs’ instructional practices with their belief systems in PE. More case studies, experimental designs, or longitudinal studies of CTs’ beliefs and practices should be conducted to gather information about their unique ways of teaching PE. Such research would assist the profession in developing the best guidelines for continuing professional development and teacher education programs. In addition, the features of how CTs value physical activity and set up a developmentally appropriate environment are important considerations in developing students’ healthy lifestyles from a long-lasting perspective. In the future, researchers should look into ways to link these factors and methods in a more practical way to improve meaningful opportunities. Ultimately, it is critical to provide continuous learning and training opportunities among teachers, which would help not only promote the quality of teaching but also ensure long-term health for students.

## Figures and Tables

**Table 1:** Description of participants.

Pseudonym	Age (Years)	Teaching	Teaching Experiences (Years)	Degree	License
Ahn-wook	46	5th grade	22	Master	Elementary School Teaching National License
Boram	34	6th grade	10	Bachelor	Elementary School Teaching National License
Cheolsoo	37	3rd grade	12	Master	Elementary School Teaching National License
Dasul	43	5th grade	18	Master	Elementary School Teaching National License
Eunhee	53	5th grade	20	Master	Elementary School Teaching National License
Hyejin	41	6th grader	14	Bachelor	Elementary School Teaching National License

## Data Availability

Not applicable.
